# Room-temperature exciton-polaritons with two-dimensional WS_2_

**DOI:** 10.1038/srep33134

**Published:** 2016-09-19

**Authors:** L. C. Flatten, Z. He, D. M. Coles, A. A. P. Trichet, A. W. Powell, R. A. Taylor, J. H. Warner, J. M. Smith

**Affiliations:** 1 Department of Materials, University of Oxford, Parks Road, Oxford OX1 3PH, United Kingdom; 2 Clarendon Laboratory, Department of Physics, University of Oxford, OX1 3PU, United Kingdom

## Abstract

Two-dimensional transition metal dichalcogenides exhibit strong optical transitions with significant potential for optoelectronic devices. In particular they are suited for cavity quantum electrodynamics in which strong coupling leads to polariton formation as a root to realisation of inversionless lasing, polariton condensation and superfluidity. Demonstrations of such strongly correlated phenomena to date have often relied on cryogenic temperatures, high excitation densities and were frequently impaired by strong material disorder. At room-temperature, experiments approaching the strong coupling regime with transition metal dichalcogenides have been reported, but well resolved exciton-polaritons have yet to be achieved. Here we report a study of monolayer WS_2_ coupled to an open Fabry-Perot cavity at room-temperature, in which polariton eigenstates are unambiguously displayed. *In-situ* tunability of the cavity length results in a maximal Rabi splitting of *ħ*Ω_Rabi_ = 70 meV, exceeding the exciton linewidth. Our data are well described by a transfer matrix model appropriate for the large linewidth regime. This work provides a platform towards observing strongly correlated polariton phenomena in compact photonic devices for ambient temperature applications.

Transition metal dichalcogenides (TMDCs) have received increased attention due to the ability to produce large, atomically flat monolayer domains with intriguing optical properties^1^,^2^,^3^,^4^,^5^,^6^. Their large exciton-binding energy leads to stable exciton formation at room temperature, narrow absorption peaks and high photoluminescence quantum yields^7^,^8^,^9^,^10^,^11^. Recently it has become possible to grow atomically thin single-crystal domains of WS_2_ by chemical vapour deposition (CVD)^2^. WS_2_, like MoSe_2_ and WSe_2_ transits from being an indirect bandgap material in bulk to having a direct bandgap as a monolayer. Owing to this direct bandgap WS_2_ interacts strongly with light: even though it is a single atomic layer with a thickness of 0.8 nm absorbance values of 0.1 and strong photoluminescence (PL) can be observed (see Fig. 1c)^8^. The valley polarisation degree of freedom^12^ and a finite Berry curvature^13^ make the material suitable for spinoptronics. In particular the large exciton binding energy of ≈0.7 eV makes monolayer WS_2_ suitable for room temperature applications^7^, enabling polariton formation at such high temperatures.

Incorporated into a microcavity with sufficiently small mode volume the two-dimensionally confined exciton couples to the photonic modes resulting in the formation of new eigenstates of the system, the polariton states, that consist of an admixture of the uncoupled states. Polaritons retain the characteristics of their constituent parts e.g. they have a degree of delocalization gained from the photon, while retaining a mass (typically ~10^−4^ that of the free electron mass^14^) and a finite scattering cross section inherited from the exciton, which gives rise to non-linear effects^15^,^16^,^17^,^18^ and strongly correlated phenomena^14^,^19^,^20^,^21^.

In this Letter we present the first study in which WS_2_ is introduced experimentally to an open microcavity to take advantage of its extraordinary optical properties for exciton-polariton formation. We make use of cavity setup which enables *in-situ* tunability of the coupling strength between the optical mode and WS_2_ excitons. Strong coupling between a photonic mode and MoS_2_ has been reported in a monolithic microcavity^22^, but suffered from poorly resolved spectral features with splittings below the exciton linewidth. At low temperatures progress with transversely confined microcavities have been made^23^,^24^, allowing polariton formation in MoSe_2_. The approach we present here entails unambiguous room temperature polariton formation with a Rabi splitting of *ħ*Ω_Rabi_ = 70 meV, exceeding the exciton linewidth and allowing *in-situ* variability of the coupling strength. We extend the theoretical description of classical polariton formation to the room-temperature large linewidth regime, for which we found corrections to current theory^25^. Furthermore we investigate the polariton distribution as a function of cavity detuning and coupling strength. Our findings establish a platform towards integrated polariton devices at room temperature suitable for spinoptronics and strongly correlated phenomena.

## Results

**Figure 1:**
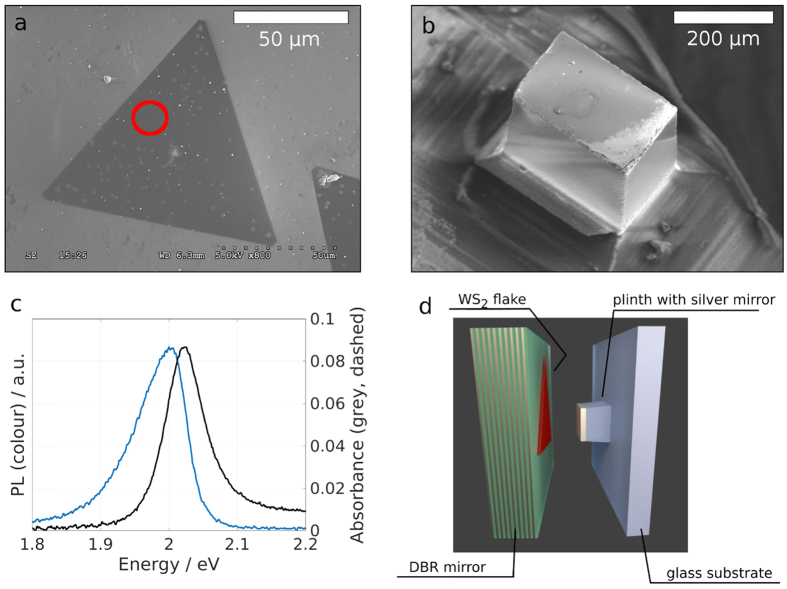
WS_2_ in an open microcavity. (**a**) SEM micrograph of a triangular monolayer WS_2_ flake transferred onto a DBR mirror. (**b**) Elevated silica plinth with a silver coating forming one side of the microcavity. (**c**) Absorbance (black, dashed) and photoluminescence (colour) spectra after off-resonant, continuous wave excitation (*λ*_exc_ = 473 nm) of marked region in **a**. (**d**) Schematics of cavity setup: The left side consists of a DBR with 10 pairs of SiO_2_/TiO_2_ with single WS_2_ flakes transferred to the low refractive-index terminated surface. The cavity is formed by positioning a silver mirror opposite the DBR.

The CVD grown WS_2_ flakes have lateral dimensions exceeding 100 μm (Fig. 1a), which are transfered with a PMMA transfer layer onto a low-index terminated distributed Bragg reflector (DBR). The opposite side of the microcavity is formed by a small silver mirror (Fig. 1b), which has a lower reflectivity than the DBR resulting in a cavity finesse of *F* ≈ 50. By placing the silver mirror opposite a region of the DBR holding WS_2_ (Fig. 1d) and varying the cavity length with a Piezo microactuator, stable cavity modes interacting strongly with the WS_2_ can be obtained (see Methods).

**Figure 2:**
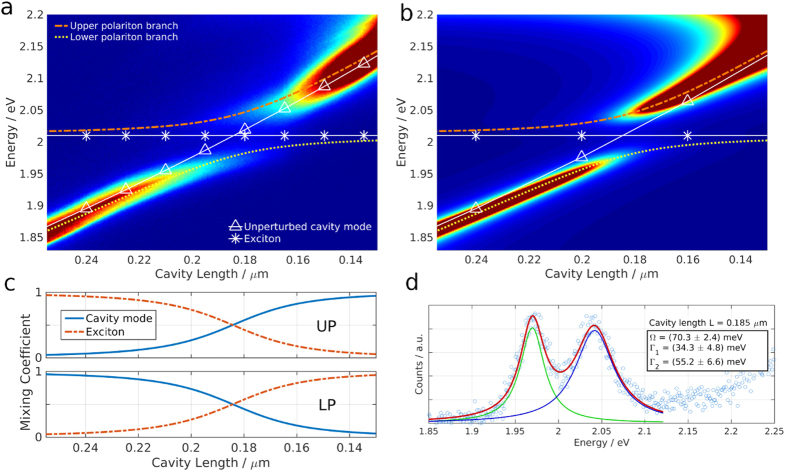
Polariton dispersion at room temperature. (**a**) Transmission spectra as the cavity length is swept, showing typical polariton dispersion. The white continuous lines show the energy of the unperturbed cavity mode (triangles) and the exciton (stars). The energy of the coupled system, the two polariton branches are shown with the coloured, dashed lines. (**b**) Transmission spectra as cavity length is swept obtained by transfer matrix modelling. The dispersion lines from (**a**) are overlaid. (**c**) Photonic and excitonic fractions of upper (UP) and lower (LP) polariton branch. (**d**) Transmission spectrum at maximal photon-exciton mixing revealing a Rabi Splitting of Ω_Rabi_ = 70 meV.

Figure 2a shows successively acquired transmission spectra for different cavity lengths tracking the mode with longitudinal mode number *q* = 3. The cavity length is decreased from left to right from 260 nm to 130 nm which leads to a linear response in cavity mode energy moving from 1.85 eV to 2.15 eV. The exciton energy of the monolayer WS_2_ stays constant at 2.01 eV (white lines in Fig. 2a). These two states are the uncoupled photon and exciton states, which couple in our system and show a typical avoided level crossing, forming the upper (UP) and lower polariton (LP) branch (coloured and dashed lines). Figure 2b shows the calculated transmission spectra obtained with a transfer matrix method (TMM). The strongly coupled system can be described by the Hamiltonian


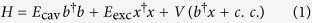


where *E*_cav_ and *E*_exc_ correspond to the energy levels of cavity mode and exciton which are coupled by the interaction potential 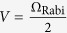. *b*^†^, *b* and *x*^†^, *x* are the respective creation and annihilation operators. The system can be reduced to:


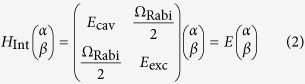


The eigenstates of the equation represent superpositions of the bare states, photonic mode and exciton, which are called polaritons. The coefficients *α*^2^ and *β*^2^ quantify the contribution of photonic and excitonic part respectively, and are plotted in Fig. 2c. As the cavity mode is tuned through the exciton energy the lower (upper) polariton branch switches from photon- (exciton)-like to exciton- (photon)-like. Figure 2d contains a vertical slice through the data presented in Fig. 2a at the crossing point of exciton and photon energy. The Rabi splitting is evaluated to *ħ*Ω_Rabi_ = (70 ± 2) meV from a fit to the data using two Lorentzian lineshapes. The splitting is fully resolved, since the individual linewidths of upper and lower polariton branch are (55 ± 7) and (34 ± 5) meV respectively, smaller than *ħ*Ω_Rabi_. Note that the cavity mode linewidth increases from ≈30 meV to ≈60 meV across the energy window presented in Fig. 2 even without the presence of an absorber due to the edge of the stop-band of the DBR which is centered around 1.95 eV (*λ* = 637 nm). The agreement between TMM data and the experimentally obtained spectra is excellent.

**Figure 3:**
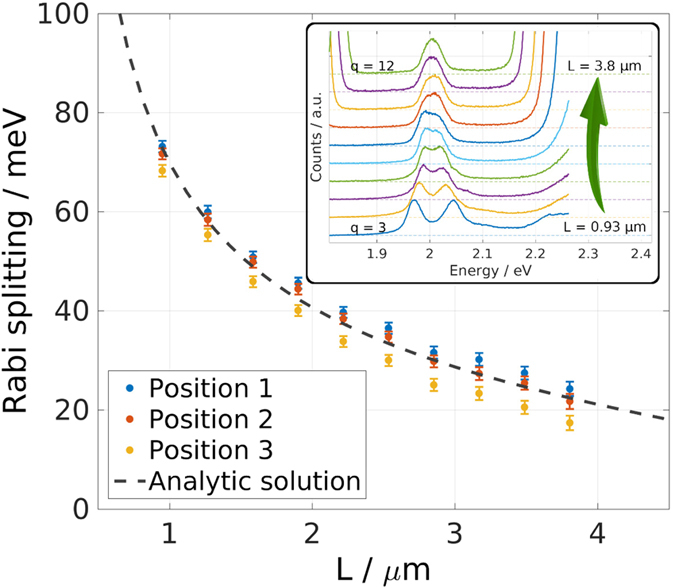
Varying the Rabi splitting. Rabi Splitting for different longitudinal cavity modes (*q* = 3, ..., 12). Symbols show experimentally obtained values for three different positions on the WS_2_ flake with corresponding uncertainty. The dashed line corresponds to the analytic expression shown in Eq. 3, derived in the Suppl. Materials. The inset depicts transmission spectra for the different longitudinal mode indices stacked vertically for better visualisation.

The open-access design of the microcavity allows for opening the cavity freely, which gives access to different longitudinal mode numbers *q*. For increasing cavity length (increasing *q*) the coupling strength between cavity mode and the WS_2_ monolayer decreases. Figure 3 presents data obtained by evaluating the polariton dispersion for the first ten accessible longitudinal mode numbers (*q* = 3, ..., 12). The two polariton branches are described by two Lorentzian peaks, whose peak positions are fitted with the dispersion obtained analytically from diagonalisation of Eq. 2. The Rabi splitting *ħ*Ω_Rabi_ is obtained as one parameter of this fit and plotted as symbols in Fig. 3. The inset in Fig. 3 displays the transmission spectra at the crossing point for the different longitudinal mode indices. The dashed line shows the analytic solution for the Rabi splitting derived by solving Maxwell’s equations for the specific cavity geometry. Thus:


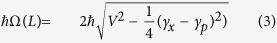



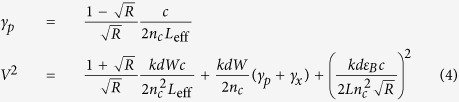


Here *R* is the mirror reflectivity (in our case *R*_Silver_ ≪ *R*_DBR_, so that we can set *R* = *R*_Silver_), *L*_*eff*_ = *L* + *L*_DBR_ (*L* is the geometric cavity length, *L*_DBR_ is the effective length of the DBR^26^), *γ*_*p*_, *γ*_*x*_ are the cavity and exciton half-widths (HWHM), *c* the speed of light, *n*_*c*_ the refractive index within the cavity, *d* the width of the monolayer (*d* = 0.8 nm), 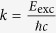, 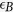 the dielectric background and *W* is a parameter proportional to the oscillator strength of the WS_2_ monolayer. We derive these expressions in the supplementary material. 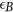 and *W* can be directly obtained from the dielectric function of monolayer WS_2_^27^, thus the system has no free parameter. For the fit to the data presented in Fig. 3 it is sufficient to measure the absorbance of the flake, which shows slight spatial variations for our sample, and obtain *W* in this way (see Fig. 3). Note that Eq. 4 has been published before for quantum wells without the second and the third term in the expression for *V*^2^ 25,26. The first correction term is small for 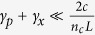, but for room temperature polariton applications it becomes sizable. In our case it corrects the value for *V*^2^ by 7% for *L* = 1 μm and by 25% for *L* = 4 μm. The second correction term can be neglected if 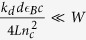, which in our system is only the case for cavity lengths exceeding *L* ≈ 2.5 μm where it contributes less than 10% (see Suppl. Information for a detailed discussion).

**Figure 4:**
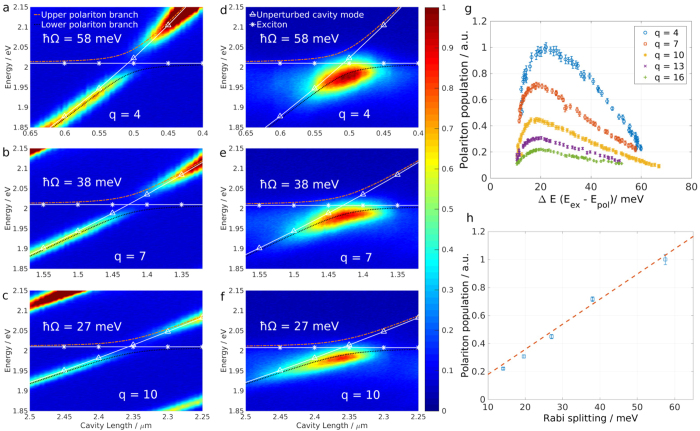
Polariton population with off-resonant pump. Transmission (**a–c**) and PL (**d–f**) spectra as cavity length is swept, traversing different longitudinal modes (*q* = 4, 7, 10). For PL measurements the sample is excited off-resonantly with a (*λ*_exc_ = 473 nm) continuous-wave laser. The lines show the dispersion of the uncoupled (white) and coupled system (colour) as in Fig. 1. (**g**) Polariton population as obtained from Lorentzian lineshape fits to the PL data for *q* = 4, 7, 10, 13 and 16. h) Maximal polariton population as a function of the Rabi splitting *ħ*Ω. Dashed line shows a linear dependence to guide the eye.

Interestingly the change in cavity length and the subsequent modulation of the Rabi splitting has stark consequences for polariton dynamics. When performing an optical transmission measurement polariton states are probed by coupling to the photonic component, which leads to the typical changes in intensity across a branch. A different way to populate exciton-polariton states is to optically pump the excitons non-resonantly, causing scattering and direct radiative pumping. Figure 4a–c display white lamp transmission data for higher *q* values *q* = 4, 7 and 10, showing a decreasing degree of coupling. The overlaid lines depict the uncoupled (white, continuous) and coupled (colour, dashed) dispersions as before (comp. Fig. 2). For the datasets presented in Fig. 4d–f the cavity length is scanned in the same way as for the transmission datasets, but here the sample is excited with a *λ* = 473 nm continuous wave laser. The resulting spectra show that only the central region of the lower polariton branch is populated. In particular the intensity and shape of the emission along the branch varies for different longitudinal mode numbers for the same laser irradiation. It is thus a function of the Rabi splitting. Note that the modulation of the incident laser power due to the change in cavity length is negligible, as the pump wavelength is far below the stopband of the mirror through which the monolayer is excited. Figure 4g shows the polariton population for *q* = 4, 7, 10, 13 and 16 as a function of the energy difference Δ*E* between polariton branch and exciton energy (Δ*E* = *E*_exc_ − *E*_LP_). It is obtained by fitting a Lorentzian lineshape to the PL data presented in Fig. 4d–f and scaling the obtained amplitudes by the inverse of the square of the photonic fraction *α* for the respective polariton branch. In general the lower polariton branch is populated slowly while its energy approaches the exciton energy from below. The population reaches a maximum between 15 meV < Δ*E* < 30 meV and decreases rapidly for Δ*E* → 0. The absolute polariton population number is not the same for different longitudinal cavity modes and decreases for a smaller Rabi splitting. In fact Fig. 4h shows the maximum polariton population as a function of the associated Rabi splitting, revealing a linear dependence. This trend could be explained by the scattering rate from exciton reservoir to polariton state, which is approximately proportional to the energy difference between the two states^28^ and governs the polariton population in the steady state. The upper polariton branch stays unpopulated for all cases.

## Discussion

The off-resonant pump leads to excitation high in the conduction band of the WS2 monolayer which is followed by a rapid thermalisation, creating an exciton bath which then populates the lower polariton branch^29^,^30^. Two known pathways for populating processes are the direct radiative decay channel, whose rate is proportional to the photonic coefficient *α* of the LPB and the phonon-assisted scattering of excitons into the LPB which is proportional to the excitonic coefficient *β*^31^. On the other hand there are multiple relaxation pathways for polaritons: the direct radiative decay proportional to *α* dominating for large Δ*E*, exciton-electron scattering proportional to *β*^32^ and exciton-exciton annihilation^33^ proportional to *β*^2^. The processes proportional to powers of *β* result in the fast decay of polaritons for small Δ*E*. The quantitative description of this system bears potential for further studies.

In conclusion we have demonstrated strong coupling between photonic cavity modes and excitons in two-dimensional atomically-thin WS_2_ at room temperature. The coherent exchange of energy between those two constituents results in the formation of exciton-polaritons with a vacuum Rabi splitting of 70 ± 3 meV. Monolayers of WS_2_ represent a promising candidate for polariton based devices due to their large exciton binding energies allowing for room temperature operation and very large oscillator strengths. We demonstate *in situ* control over the coupling strength and derive an analytic expression describing the length dependence. By studying the PL of the device we observe that the coupling strength directly influences the population dynamics.

Strongly coupled devices of the type described here could provide a route towards observing strongly correlated phenomena at room temperature. In particular properties such as the valley polarisation degree of freedom^12^ and a finite Berry curvature^13^ render devices based on TMDCs attractive for spinoptronics and quantum computation.

## Methods

### Sample preparation

The open microcavity consists of two opposing flat mirrors, a large dielectric DBR with 10 pairs of SiO_2_, TiO_2_ with central wavelength of *λ* = 637 nm and a smaller silver mirror with a thickness of 50 nm, deposited via thermal evaporation (Fig. 1). The WS_2_ flakes are transferred onto the dielectric mirror stack, which has a low refractive-index terminated configuration to provide an anti-node of the electric field at the mirror surface and thus optimal coupling to the monolayer. Note that this condition rules out the recently described Tamm-plasmonic coupling, as it requires a high refractive-index termination before the metallic layer^34^. This transfer process is facilitated by coating the as-grown WS_2_ flakes on SiO_2_ with a helper layer of PMMA. After etching away the substrate, the floating PMMA film was transfered manually onto the DBR and baked at 150° for 15 min. The remaining PMMA was then dissolved by placing the sample in an acetone bath for 10 min. After non-resonant excitation with a *λ* = 473 nm laser the WS_2_ flakes show strong neutral exciton emission at 2.01 eV with little, spatially varying contribution from the charged exciton state at 1.98 eV (see Fig. 1c), which we attribute to a non-uniform excess electron background^7^. The small silver mirror is mounted on a three-dimensional piezo actuated stage, which makes positioning of the silver mirror relative to the WS_2_ flake possible and allows for electrical control of the cavity length.

### Optical measurements

By positioning the silver plinth over a region of the DBR mirror which holds monolayer WS_2_ and reducing the distance between the two mirrors below ≈5 μm, stable cavity modes interacting strongly with the WS_2_ excitons will appear in a transmission experiment. The light is focused onto an Andor combined spectrometer/CCD for analysis. The setup allows for off-resonant optical excitation below the stop-band of the DBR with a continuous wave laser with wavelength *λ* = 473 nm at power densities around 
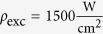
.

## Additional Information

**How to cite this article**: Flatten, L. C. *et al*. Room-temperature exciton-polaritons with two-dimensional WS_2_. *Sci. Rep.* **6**, 33134; doi: 10.1038/srep33134 (2016).

## Supplementary Material

Supplementary Information
