# Supplementary material for: Room-temperature exciton-polaritons with two-dimensional WS2
Source: Sci Rep. 2016 Sep 19;6:33134. doi: 10.1038/srep33134 (PMC5027543; doi:10.1038/srep33134)
Supplement: Supplementary Information [file srep33134-s1.pdf]

Supplemental Materials: Room-temperature exciton-polaritons with two-dimensional WS_2

L. C. Flatten,[1,*] Z. He,[1] D. M. Coles,[1,2] A. A. P. Trichet,[1]
A. W. Powell,[1] R. A. Taylor,[2] J. H. Warner,[1] and J. M. Smith[1]

[1]*Department of Materials, University of Oxford, Parks Road, Oxford OX1 3PH, United Kingdom*
[2]*Clarendon Laboratory, Department of Physics, University of Oxford, OX1 3PU, United Kingdom*

[*] lucas.flatten@materials.ox.ac.uk

Appendix A: Additional information

The data presented in this work are available at https://ora.ox.ac.uk:443/objects/uuid:d3f0b229-df66-4895-ba1e-0d9ef9a07ec9. During the publication process, the authors became aware of a related study by Wang et al. [S1], demonstrating exciton-polaritons with monolayer WS2 incorporated into a monolithic metallic cavity.

Appendix B: Details of experimental setup

The DBR mirror consists of 10 pairs of SiO_2, TiO_2 with refractive indices 1.45 and 2.05 respectively and the stopband is centered around $\lambda = 637\,\mathrm{nm}$. The small mirror is produced by removing large areas of a flat silica substrate with a dicer to create a $200 \times 300\ \mu\mathrm{m}^2$ plinth made semi-reflective by thermally evaporating a 50 nm thick silver layer with a reflectivity of $R = 95\%$. It is mounted on a three-dimensional piezo-actuated stage, which makes electronic positioning of the silver mirror relative to the WS_2 flake possible. To initialise the cavity white light from a light emitting diode is shone through the mirrors while reducing the separation. With the help of Fabry-Perot fringes visible for small mirror separations ($L < 20\,\mu\mathrm{m}$) the substrates are made parallel within 150 µrad. Optical access to the sample is given by a standard $\times 10$ objective lens and the collected light is focused on an Andor combined spectrograph/CCD with a 300 grooves/mm grating. For the photoluminescence experiment the sample is excited with a PicoQuant D-C-470 laser with $\lambda = 473\,\mathrm{nm}$ at power densities around $\rho_{\mathrm{exc}} = 1500\frac{\mathrm{W}}{\mathrm{cm}^2}$.

Appendix C: Derivation of analytic expression for Rabi splitting

1. Transfer matrices

Maxwell's equation in a spatially homogeneous medium with refractive index n reduce to

$$\nabla^2 \vec{E} + k^2 n^2 \vec{E} = 0 \tag{S1}$$

with the vacuum wavenumber $k = \frac{2\pi}{\lambda}$. In particular we get $\partial_z^2 E = -k^2 n^2 E$ for the field amplitude in z-direction. A general form of the solution for waves in this direction is

$$E(z) = A^+ e^{inkz} + A^- e^{-inkz} \tag{S2}$$

A^+ and A^- are the amplitudes of forward and backward travelling waves. For the transition from a medium with refractive index n_1 to one with n_2 we can define the amplitude reflection and transmission coefficients:

$$r = \frac{A_1^-}{A_1^+} \qquad \& \qquad t = \frac{A_2^+}{A_1^+} \tag{S3}$$

To solve the boundary conditions for multiple interfaces it is convenient to define a transfer matrix T, which propagates the wave across a homogeneous layer. The analytic form of such a matrix can be obtained after chosing a convenient basis set. One possibility is to define the vector [S2]

$$\Phi(z) = \begin{pmatrix} E(z) \\ \frac{1}{ik}\partial_z E(z) \end{pmatrix} \tag{S4}$$

A matrix that enforces the propagation through a layer with refractive index n and thickness a, $T_a \Phi|_{z=0} = \Phi|_{z=a}$ has the form

$$T_a = \begin{pmatrix} \cos(nka) & \frac{i}{n}\sin(nka) \\ in\sin(nka) & \cos(nka) \end{pmatrix} \tag{S5}$$

2. Application to dispersive medium in planar cavity

We reduce the problem of finding the resonant modes in the cavity to finding the amplitude reflection coefficient r as defined in Fig. S1. Having found r we can compute the cavity dispersion by solving ($k_c = n_c k$)

$$\begin{pmatrix} \cos(k_c L) & \frac{i}{n_c}\sin(k_c L) \\ in_c\sin(k_c L) & \cos(k_c L) \end{pmatrix} \begin{pmatrix} 1 + r_1 \\ n_c(r_1 - 1) \end{pmatrix} = A \begin{pmatrix} 1 + r \\ n_c(1 - r) \end{pmatrix} \tag{S6}$$

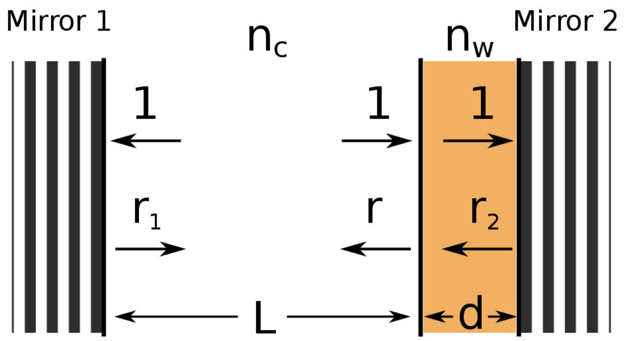

FIG. S1. **A thin dispersive medium inside a planar microcavity.** Schematics of planar cavity with mirror separation $L + d$ and refractive index n_c for the cavity medium and n_w for the thin dispersive medium. The arrows give the amplitudes of forwards and backwards propagating waves as introduced in the text.

The choice of coefficients for the vectors on each side of the cavity ensures the correct reflection conditions, i.e. attenuation and phase jump. Eliminating A we obtain

$$r_1 r e^{i2k_c L} = 1 \tag{S7}$$

For ideal dielectric mirrors we would have $r_1 = r = 1$ and thus the well known Fabry-Perot resonance condition $k_c L = \pi q$ emerges, q being an integer. Now the problem is reduced to finding r. Taking the right side of the sketch in Fig. S1 we can start with $(k_w = n_w k)$

$$\begin{pmatrix} \cos(k_w d) & \frac{i}{n_w}\sin(k_w d) \\ i n_w \sin(k_w d) & \cos(k_w d) \end{pmatrix} \begin{pmatrix} 1+r \\ n_c(1-r) \end{pmatrix} = A \begin{pmatrix} 1+r_2 \\ n_w(1-r_2) \end{pmatrix} \tag{S8}$$

Eliminating A and solving for r we get:

$$r = \frac{n_c - n_w + e^{i2k_w d}(n_c + n_w) r_2}{n_c + n_w + e^{i2k_w d}(n_c - n_w) r_2} \tag{S9}$$

This expression is further reducible if we assume a certain form for r_2. A suitable choice would be the reflection coefficient for a distributed Bragg reflector r_{DBR} or metallic mirror r_{Met} (derived in [S2]):

$$r_{\text{DBR}} = \sqrt{R} e^{i(k - k_{\text{DBR}})\frac{L_{\text{DBR}}}{n_w}} \qquad \& \qquad r_{\text{Met}} = \frac{n_w - n_m}{n_w + n_m} \tag{S10}$$

Here R is the mirror reflectivity, $L_{\text{DBR}} = \frac{n_a n_b \lambda_{\text{DBR}}}{2(n_b - n_a)}$ is the effective length of the DBR, with λ_{DBR} the central wavelength, $k_{\text{DBR}} = \frac{2\pi}{\lambda_{\text{DBR}}}$ and n_m the complex refractive index of the metal. If we assume a perfect dielectric reflector $(r_2 = 1)$ we obtain:

$$r = \frac{n_c \cos(k_w d) + i n_w \sin(k_w d)}{n_c \cos(k_w d) - i n_w \sin(k_w d)} \tag{S11}$$

Substituting this expression into Eq. S7, assuming a thin layer $(|k_w d| << 1)$ and setting $r_1 = 1$ we obtain:

$$n_c \sin(k_c L) + n_w k_w d \cos(k_c L) = 0 \tag{S12}$$

Evaluating this expression close to the cavity mode resonance $(\cos(k_c L) \approx 1, \sin(k_c L) \approx n_c(k - k_p)L$, with k_p being the wavenumber on resonance) we get to the succinct expression:

$$n_c^2 (k - k_p) L + n_w^2 k d = 0 \tag{S13}$$

Now we want to substitute in n_w, the complex refractive index of the dispersive thin layer, and find the dispersion of the resonant modes. The dielectric function of a system of Lorentz oscillators as derived from solving the optical Bloch equations can be written as [S3]

$$\varepsilon(k) = \varepsilon_{\text{B}} - \frac{W}{c(k - k_x) + i\gamma_x} \tag{S14}$$

where W is proportional to the oscillator strength of the material, k_x is the wavenumber of the oscillator resonance and γ_x is the oscillator HWHM linewidth. The relation between W and microscopic material parameters is derived in [S4] and reads

$$W = \frac{N\mu^2}{3\varepsilon_0 \hbar V} \tag{S15}$$

with $\frac{N}{V}$ the number of oscillators per volume and μ the transition dipole moment for a single oscillator. From now on we set $\hbar = c = 1$. Putting $n_w = \sqrt{\varepsilon(k)}$ into Eq. S13 we obtain:

$$n_c^2(k - k_p + i\gamma_p)L + \left(\varepsilon_{\mathrm{B}} - \frac{W}{k - k_x + i\gamma_x}\right) kd = 0 \tag{S16}$$

At this stage we have included a term $i\gamma_p$ for the finite linewidth of the cavity mode. Of course this is artifical at this point and we will derive γ_p in terms of the reflectivity R of the mirrors later. We assume that k varies little over the extent of the thin dispersive layer, in other words the factor kd is constant ($kd = k_d d$). We can then solve the equation for k and find by neglecting higher orders in d:

$$k_{1,2} = \frac{1}{2}\left(k_p + k_x - i(\gamma_p + \gamma_x)\right) \pm \sqrt{\frac{Wk_d d}{Ln_c^2} + \frac{1}{4}(k_p - k_x + i(\gamma_x - \gamma_p))^2)} \tag{S17}$$

If the radicand is positive, these are the solutions for the two polariton branches. Commonly one sets $V^2 = \frac{Wk_d d}{Ln_c^2}$, evaluates the system on resonance ($k_p = k_x$) and compares the terms of the radicand. Then $2V < |\gamma_x - \gamma_p|$ marks the weak coupling regime, in which no mode splitting occurs and $2V > |\gamma_x - \gamma_p|$ falls into the strong coupling regime with finite normal mode splitting. Note that the Rabi splitting $\Omega = 2\sqrt{V^2 - \frac{1}{4}(\gamma_x - \gamma_p)^2)}$ only follows V if the linewidths can be neglected in the radicand. As well note that a system given by two coupled, damped oscillators as:

$$(k - k_p + i\gamma_p)(k - k_x + i\gamma_x) = V^2 \tag{S18}$$

has solutions of the form Eq. S17 with V as given above.

Now the question is, what shape the artificially introduced cavity linewidth γ_p has in terms of the mirror reflectivity. To this end we set $r_1 = \sqrt{R}$ and follow the above procedure again. We find lengthy solutions for $k_{1,2}$ in which we can identify

$$\begin{aligned}
\gamma_p &= \frac{1-\sqrt{R}}{\sqrt{R}}\frac{1}{2n_cL} \\
V^2 &= \frac{k_d dW}{4n_c^2 L}\left(\frac{3+\sqrt{R}}{\sqrt{R}} + 2Ln_c\gamma_x\right) + \left(\frac{k_d d\epsilon_B}{2Ln_c^2\sqrt{R}}\right)^2 \\
&= \frac{1+\sqrt{R}}{\sqrt{R}}\frac{k_d dW}{2n_c^2 L} + \frac{k_d dW}{2n_c}(\gamma_p + \gamma_x) + \left(\frac{k_d d\epsilon_B}{2Ln_c^2\sqrt{R}}\right)^2
\end{aligned} \tag{S19}$$

In this we have neglected terms of higher order in d, except for one term proportional to ϵ_B^2, and assumed $k_d d\epsilon_B \ll n_c$. Comparing these solutions with expressions found by Savona et al. [S5]

$$\gamma_{p_{\mathrm{Sav}}} = \frac{1-\sqrt{R}}{\sqrt{R}}\frac{1}{n_cL} \qquad \& \qquad V_{\mathrm{Sav}}^2 = \frac{1+\sqrt{R}}{\sqrt{R}}\frac{\Gamma_0}{n_cL} \tag{S20}$$

we identify a few differences. Here Γ_0 is proportional to the oscillator strength of the quantum well. γ_p matches the published one, the difference in the denominator stems from the different cavity geometry. The published form of V^2 lacks the two last terms in our truncated solution [S2, S5]. The first correction term $\frac{k_d dW}{2n_c}(\gamma_p + \gamma_x)$is small for small linewidths $\gamma_p + \gamma_x \ll \frac{2c}{n_cL}$, but for room temperature polariton applications it becomes sizable. In our case it corrects the value for V^2 by 7% for $L = 1$ µm and by 25% for $L = 4$ µm. The second term $\left(\frac{k_d d\epsilon_B}{2Ln_c^2\sqrt{R}}\right)^2$ is small for large cavity lengths and low refractive indices of the thin dispersive medium, i.e. for $\frac{k_d d\epsilon_B^2}{4Ln_c^2} \ll W$. In our case, where $\epsilon_B = 21$ is large [S6], it adds about 28% for $L = 1$ µm and 7% for $L = 4$ µm to the value of V^2 (see Fig. S2 inset).

Fig. S2 shows the difference of these expressions. The black dashed line shows $\Omega \propto \frac{1}{\sqrt{L}}$, the blue continuous line depicts the analytic solution without truncation of terms in higher order of d, the red dashed line corresponds to our truncated solutions from Eq. S19 and the yellow dashed line shows the solution from Savona et al. [S5] (Eq. S20). Parameters are chosen as for the fit to our data. In particular we chose $W = 21\gamma_x = 588\,\text{meV}$. We have included the experimental data from one position from the main text to demonstrate the difference in slope and magnitude to the solution given by Eq. S20. Note that our model predicts a larger coupling for small cavity lengths, in particular we estimate a Rabi splitting of ≈ 180 meV for a $L = \frac{\lambda}{2} = 310$ nm cavity.

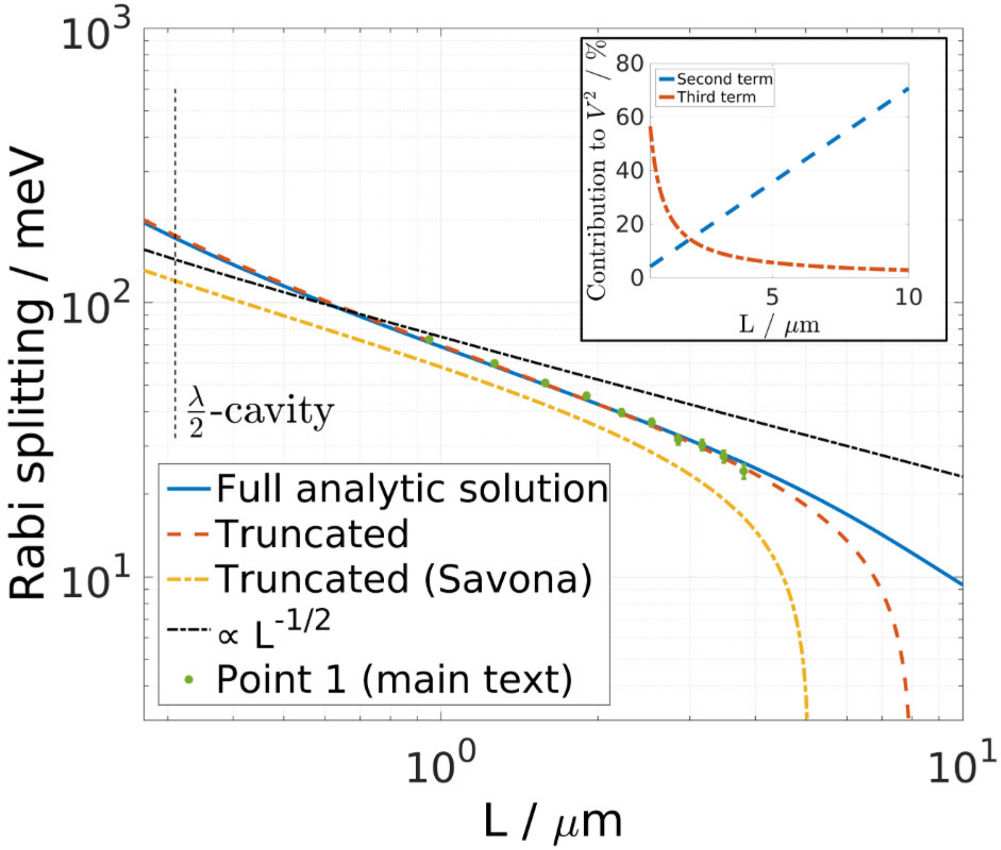

FIG. S2. **Rabi splitting Ω as obtained by different analytical expressions.** The black dashed line shows $\Omega \propto \frac{1}{\sqrt{L}}$, the blue continuous line depicts the analytic solution without truncation of terms in higher order of d, the red dashed line corresponds to our truncated solutions from Eq. S19, the yellow dashed line shows the solution from Savona et al. [S5] (Eq. S20). The green dots are experimental values as presented in the main text. Parameters have the numeric value: $\epsilon_B = 20$, $\gamma_x = 28\,\text{meV}$, $R = 0.95$, $k_x = 2\,\text{eV}$, $k_p = 2\,\text{eV}$, $d = 0.8\,\text{nm}$, $n_c = 1$, $W = 21\gamma_x$ (see [S6] for choice of ϵ_B & W).

Appendix D: Equivalence between phenomenological Hamiltonian and TMM approach for description of polariton dispersion

In this work we have used two different descriptions for the strongly coupled system of a cavity mode and the monolayer of WS_2. Here we show the equivalence between both descriptions.

In the main text we have limited our description to a single cavity mode with energy E_c and a single exciton state E_x, coupled by a phenomenologically introduced interaction with strength $\frac{\hbar\Omega_{\text{Rabi}}}{2}$. The system given by Eq. (1) is solved by using the Hopfield transformation resulting in mixed cavity-exciton states with lower and upper polariton operators. Eq. (2) describes the same system in a basis representation, here in the basis of cavity mode $|\alpha\rangle$ and exciton state $|\beta\rangle$. The eigenvalues of this matrix are given by:

$$E_{1,2} = \frac{1}{2}(E_{\text{cav}} + E_{\text{exc}}) + \frac{1}{2}\sqrt{(E_{\text{cav}} - E_{\text{exc}})^2 + \Omega_{\text{Rabi}}^2} \qquad \text{(S21)}$$

which is equivalent to Eq. S17 when neglecting the linewidths of both systems. Indeed the relation $\Omega_{\text{Rabi}} = 2V$ is motivated by this equivalence. We thus see, that both the phenomenological Hamiltonian in Eq. (1) and the TMM approach, which is based on classical electromagnetism and a simple oscillator model, results in the same dispersion. Other parameters, such as the polariton linewidths, are only explained by the classical model. For a more extensive description of strongly coupled systems we point the reader to the literature: Kavokin and coworkers give a comprehensive review of polariton formation within the framework of three different formalisms: the classical, the semiclassical and the full quantum description [S2]. Savona et. al. have described the polariton formation in quantum well microcavities with both a semiclassical TMM approach and a quantum theoretical approach [S5]. Equations from both approaches (Eq. (7) and (10) in Ref. [S5]) give the same result which we have found in Eq. S18. While Savona et al. start off by supposing quantised radiation modes in their Hamiltonian Eq. (8) in Ref. [S5], a more rigorous derivation entailing quantisation of both the photon field and the exciton state is given by Gerace et al. [S7].

[S1] Wang, S. *et al.* Coherent Coupling of WS_2 Monolayers with Metallic Photonic Nanostructures at Room Temperature. *Nano Letters* **16**, 4368–4374 (2016).

[S2] Kavokin, A., Baumberg, J. J., Malpuech, G. & Laussy, F. P. *Microcavities* (OUP Oxford, Oxford ; New York, 2011), revised ed. edn.

[S3] Khitrova, G., Gibbs, H. M., Jahnke, F., Kira, M. & Koch, S. W. Nonlinear optics of normal-mode-coupling semiconductor microcavities. *Reviews of Modern Physics* **71**, 1591–1639 (1999).

[S4] Loudon, R. *The Quantum Theory of Light* (Oxford University Press, 2000), third edn.

[S5] Savona, V., Andreani, L. C., Schwendimann, P. & Quattropani, A. Quantum well excitons in semiconductor microcavities: Unified treatment of weak and strong coupling regimes. *Solid State Communications* **93**, 733–739 (1995).

[S6] Li, Y. *et al.* Measurement of the optical dielectric function of monolayer transition-metal dichalcogenides: MoS_2, $MoSe_2$,WS_2 and WSe_2. *Physical Review B* **90**, 205422 (2014).

[S7] Gerace, D. & Andreani, L. C. Quantum theory of exciton-photon coupling in photonic crystal slabs with embedded quantum wells. *Physical Review B* **75**, 235325 (2007).
